# Hypoglycemic Effects of *Glehniae Radix* Polysaccharides in Female *db*/*db* Mice via Modulation of the Gut Microbiota-PPAR Signaling Axis

**DOI:** 10.3390/molecules31061046

**Published:** 2026-03-20

**Authors:** Haochen Xu, Hanqing Lin, Hetong Lin, Peng Wu, Fang Zhang, Longhe Yang

**Affiliations:** 1 College of Food Science, Fujian Agriculture and Forestry University, Fuzhou 350002, China; xuhaochen1008@163.com (H.X.); papapu.ppa.pu@gmail.com (H.L.); hetonglin@163.com (H.L.); 2 Technical Innovation Center for Utilization of Marine Biological Resources, Third Institute of Oceanography, Ministry of Natural Resources, Xiamen 361000, China; pwu@tio.org.cn

**Keywords:** *Glehniae Radix* polysaccharides, diabetes mellitus, gut microbiota, PPAR signaling pathway

## Abstract

*Glehniae Radix*, the dried root of *Glehnia littoralis* Fr. Schmidt ex Miq. (Apiaceae), exhibits diverse biological activities. However, research on the hypoglycemic effects of *Glehniae Radix* polysaccharides (GRPs), particularly in vivo studies clarifying their mechanisms of action, remains limited. This study aimed to verify the in vivo hypoglycemic activity of crude GRP in a diabetic model and to elucidate its mechanism. GRP was extracted by water extraction and ethanol precipitation, yielding an extraction rate of 38% and a polysaccharide content of 73.48%. Its hypoglycemic effects and mechanisms were investigated in female B6.BKS(D)-Leprdb/J (*db*/*db*) mice following daily administration of GRP at doses of 300 and 600 mg/kg for five consecutive weeks. Following GRP administration, mice in the CH group (600 mg/kg) exhibited a mean reduction in fasting blood glucose levels of approximately 40.7% and an improvement in insulin tolerance test (ITT) outcomes by about 28.4%. Additionally, GRP alleviated damage to the liver, kidney, and colon; decreased hepatic total cholesterol (TC) and triglycerides (TGs) by approximately 40.8% and 24.6%, respectively; and increased colonic Zonula Occludens-1 (ZO-1) expression by an average of 49.5%. Mechanistically, GRP significantly upregulated the expression of peroxisome proliferator-activated receptor-α (PPAR-α) and PPAR-γ in the liver, while also increasing the abundance of beneficial gut bacteria such as *Alistipes* and suppressing harmful bacteria including *Escherichia-Shigella*. Furthermore, GRP activated the galactose metabolism pathway and inhibited abnormal enrichment of the steroid biosynthesis pathway, collectively ameliorating glucose metabolic disorders in female *db*/*db* mice via the “gut microbiota–PPAR signaling axis”. In light of these results, GRP exerts significant in vivo hypoglycemic effects by modulating gut microbiota balance and activating the PPAR signaling pathway.

## 1. Introduction

With the continuous improvement of living standards and the rapid development of society and economy, the prevalence of diabetes mellitus (DM), especially type 2 diabetes (T2DM), has been increasing annually. T2DM accounts for more than 96% of all diabetes cases [1], which is largely attributed to factors such as high-sugar/high-fat (HSHF) diets. This chronic metabolic disorder is mainly characterized by insufficient insulin secretion from pancreatic β-cells, presenting primarily as insulin resistance (IR), persistent hyperglycemia, metabolic dysregulation, and impaired intestinal barrier [2,3,4,5]. Current therapeutic strategies for T2DM include medications like metformin, sulfonylureas, and thiazolidinediones. However, these synthetic drugs are often limited by side effects and drug resistance [6]. Therefore, screening and developing novel bioactive components from natural products with hypoglycemic potential hold significant research value.

*Glehniae Radix*, the dried root of *Glehnia littoralis* Fr. Schmidt ex Miq. (Apiaceae) (The plant nomenclature follows *Glehnia littoralis* (A.Gray) F.Schmidt ex Miq., Ann. Mus. Bot. Lugduno-Batavi 3: 61. 1867) [7], is a marine medicinal plant recorded in the “Marine Chinese Materia Medica” and has a long history of use as both a food and medicine, possessing a rich profile of pharmacological properties [8,9]. Due to its biological effects, *Glehniae Radix* has been widely used in traditional medicine, and modern clinical practice has also confirmed its application in the treatment of metabolic diseases such as diabetes [10]. However, the specific active ingredients of *Glehniae Radix* in the treatment of diabetes remain unclear, polysaccharides represent a major class of constituents in *Glehniae Radix* and are closely associated with its bioactivities.

Polysaccharides are natural bioactive macromolecules that exhibit a variety of pharmacological effects, including anti-inflammatory [11], anti-aging [12], and regulation of glucose and lipid metabolism [13]. Recent studies have shown that polysaccharides can significantly modulate the gut microbiota, repair the intestinal barrier [14,15,16,17,18,19]. *Glehniae Radix* polysaccharides (GRPs) are commonly extracted and purified by methods such as hot-water extraction followed by ethanol precipitation. For instance, Jing isolated a homogeneous glucan, GLP80-1, from *Glehniae Radix*, whose structure was determined to consist of a main chain of (1→4)-linked-α-D-Glcp with a single α-D-Glcp branch substituted at C-6 [20]. In vitro bioactivity assays have demonstrated that GLP and GLP80-1 exhibit activities such as free radical scavenging and immunomodulation. Previous research has indicated that the aqueous extract from *Glehniae Radix* can effectively inhibit lipid accumulation in 3T3-L1 preadipocytes and in high-fat diet-induced obese mice [21,22]. but the specific active constituents were not investigated. A systematic evaluation of the effects of GRP on blood glucose and lipid metabolism in diabetic mice is still lacking.

B6.BKS(D)-Leprdb/J (*db*/*db*) mice represent one of the most classical and widely utilized animal models for the study of T2DM and obesity. Studies have demonstrated significant sexual dimorphism in the diabetic phenotype within this model: female *db*/*db* mice exhibit distinct physiological characteristics compared to males in terms of insulin resistance progression, adipose distribution patterns, hepatic lipid metabolism, and gut microbiota composition, these differences may influence the efficacy and mechanisms of pharmacological interventions [23,24]. Furthermore, existing research has employed female *db*/*db* mice in metabolic disease studies, demonstrating relatively stable parameters and suggesting a limited impact from fluctuations in female hormones [25]. However, the vast majority of current studies evaluating polysaccharides for T2DM amelioration have focused on male animals, leaving a critical gap in systematic research regarding the hypoglycemic effects and gut microbiota modulation of natural polysaccharides in female *db*/*db* mice.

Based on this rationale, the present study selected female *db*/*db* mice as the experimental model to investigate the ameliorative effects of GRP on T2DM and to elucidate its underlying mechanisms. This work will not only provide novel insights into the potential of *Glehniae Radix* polysaccharides for the prevention and treatment of T2DM but also furnish essential data to support research on polysaccharides in female *db*/*db* mice.

## 2. Results

### 2.1. Chemical Composition Analysis and Structural Characterization of GRP

**Figure 1:**
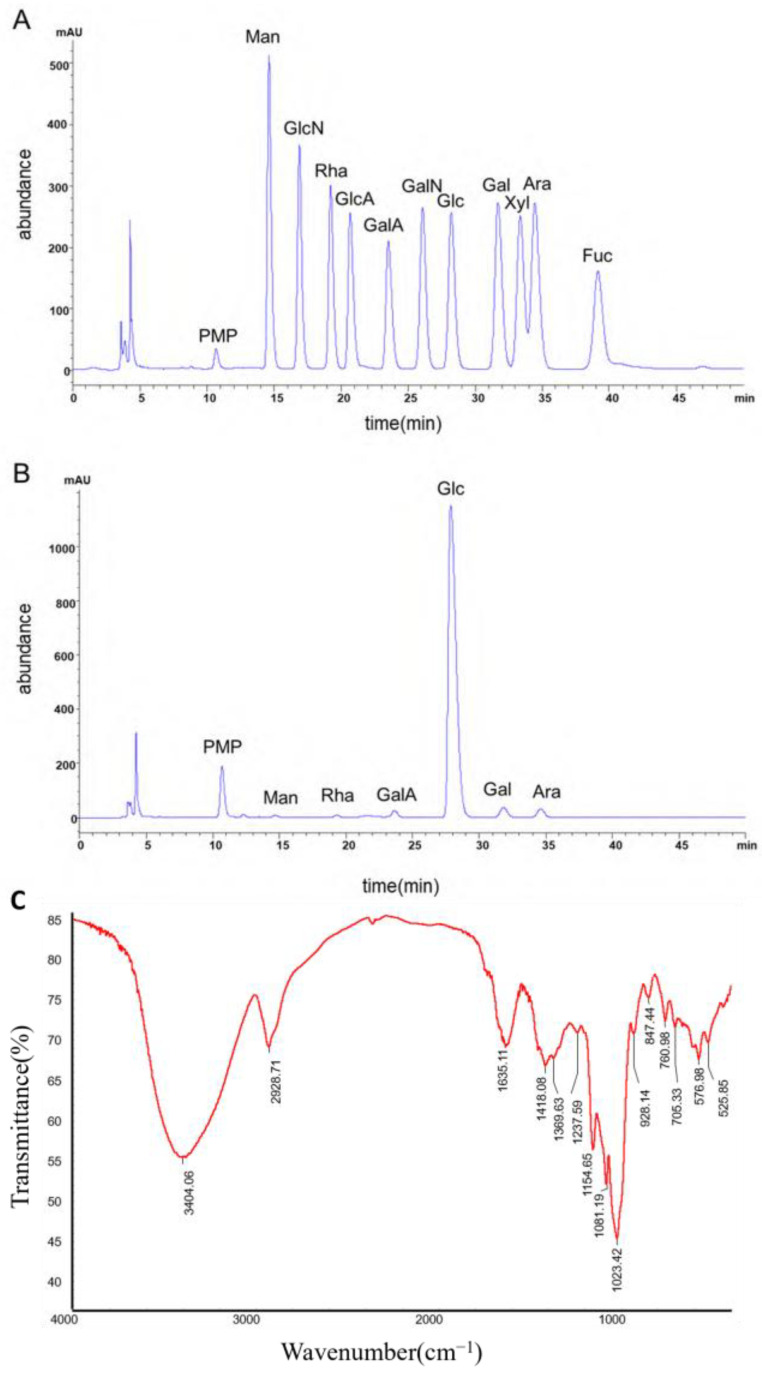
Composition and structure analysis of GRP. (**A**) High Performance Liquid Chromatography of Mixed Monosaccharide Standards. (**B**) High Performance Liquid Chromatography of GRP. (**C**) Infrared spectrum of GRP. (PMP, 1-Phenyl-3-methyl-5-pyrazolone; Man, D-Mannose; GlcN, D-Glucosamine; Rha, L-Rhamnose; GlcA, D-Glucuronic acid; GalA, D-Galacturonic acid; GalN, D-Galactosamine; Glc, D-Glucose; Gal, D-Galactose; Xyl, D-Xylose; Ara, L-Arabinose; Fuc, L-Fucose).

GRP was obtained from *Glehniae Radix* via water extraction and ethanol precipitation with a yield of 38.85%, and was characterized as primarily polysaccharides. Compositional analysis revealed a total polysaccharide content of 73.48 ± 2.85%, an acidic sugar content of 46.09 ± 1.56%, and a relatively low protein content of 0.85 ± 0.05% (Table S2), indicating minimal proteinaceous impurities. Based on the standard curve and the retention time of the major peak (11–16 min) in the molecular weight distribution profile (Figure S2), the number-average molecular weight (Mn) and weight-average molecular weight (Mw) of GRP were calculated to be 1.938 × 10^6^ Da and 4.711 × 10^6^ Da, respectively (Table S3). As shown in Figure 1A,B and Table S4, monosaccharide composition analysis revealed that GRP is predominantly composed of glucose (92.87%), followed by galactose (2.36%), arabinose (2.28%), and galacturonic acid (1.77%).

The Fourier Transform Infrared Spectroscopy (FT-IR) spectrum of GRP (Figure 1C) revealed absorption bands characteristic of polysaccharides. These included a broad band for O–H stretching at 3404 cm^−1^, a weak band for C–H stretching at 2929 cm^−1^, and a band at 1635 cm^−1^ corresponding to the C=O stretching of uronic acids. Peaks at 1154, 1081, and 1023 cm^−1^ were assigned to the C–O–C and C–O–H vibrations of the glycosidic bonds and pyranose rings. Additionally, the signals at 928 cm^−1^ and 847 cm^−1^ are characteristic of α- and β-glycosidic linkages, respectively, indicating that GRP is a heteroglycan containing both configurations.

### 2.2. GRP Ameliorates Fasting Blood Glucose and Insulin Tolerance in Female db/db Mice

**Figure 2:**
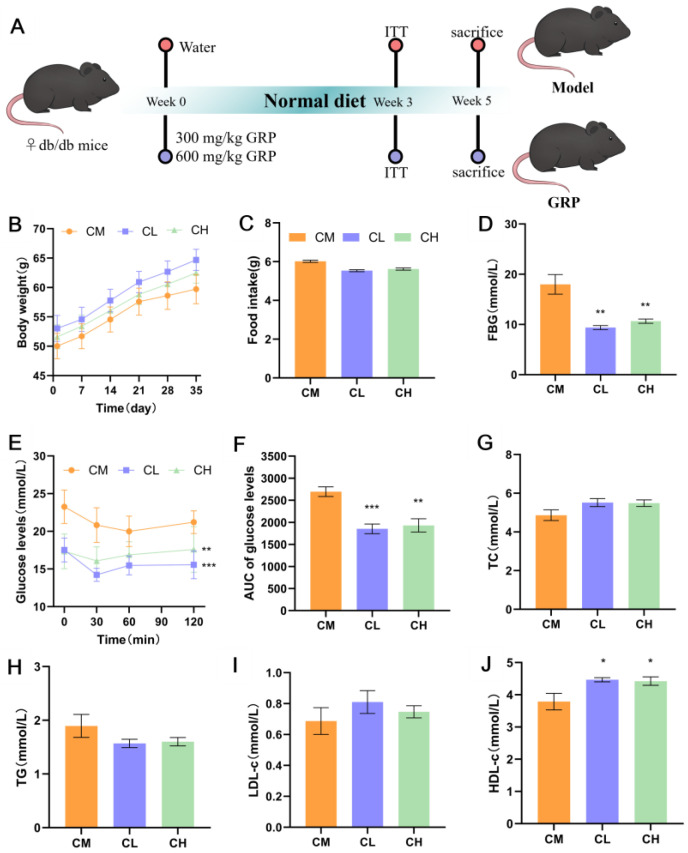
GRP reduces blood glucose and elevated HDL-c levels in serum of female *db*/*db* mice. (**A**) The experimental flow chart. Mice were experimented with Model group, GRP (300 mg/kg), GRP (600 mg/kg). (**B**,**C**) Average daily food intake and body weight of mice in different groups. (**D**) FBG levels of mice. (**E**) The curve of ITT at last treatment week. (**F**) Areas under curve (AUC) for ITT during the final week of treatments. (**G**–**J**) Serum levels of TC, TG, LDL-C, HDL-C. (* *p* < 0.05, ** *p* < 0.01, *** *p* < 0.001, Compared with CM group).

During the 5-week experimental period (Figure 2A), body weight and food intake did not differ significantly between the model (CM) and GRP-treated (CL, CH) groups (Figure 2B,C). GRP administration significantly lowered fasting blood glucose (FBG) by 47.78% and 40.66% in the CL and CH groups, respectively (*p* < 0.01; Figure 2D), and improved systemic insulin sensitivity, as evidenced by lower blood glucose levels during an insulin tolerance test (ITT) and a reduced area under the curve (AUC) by 31.24% and 28.35% (Figure 2E,F). These results demonstrate that GRP effectively ameliorates hyperglycemia and insulin resistance in female *db*/*db* mice. GRP treatment did not significantly alter serum levels of total cholesterol (TC), triglyceride (TG), or low-density lipoprotein cholesterol (LDL-c) but notably increased high-density lipoprotein cholesterol (HDL-c) levels in both treatment groups (Figure 2G–J). These results demonstrate that the improving effect of GRP on the blood lipid profile of female *db*/*db* mice was limited.

GRP restored insulin sensitivity and reduced serum glucose in female *db*/*db* mice in a dose-dependent manner, significantly improving glucose metabolism. Meanwhile, a weak regulatory effect and trend of GRP on the blood lipid profile were observed in the results of serum TG and HDL-C. It indicates that in the serum analysis, the regulatory effect of GRP on glucose metabolism was greater than that on lipid metabolism in female *db*/*db* mice.

### 2.3. GRP Amelioration Hepatic Injury and Reduction TC/TG Levels in Female db/db Mice

**Figure 3:**
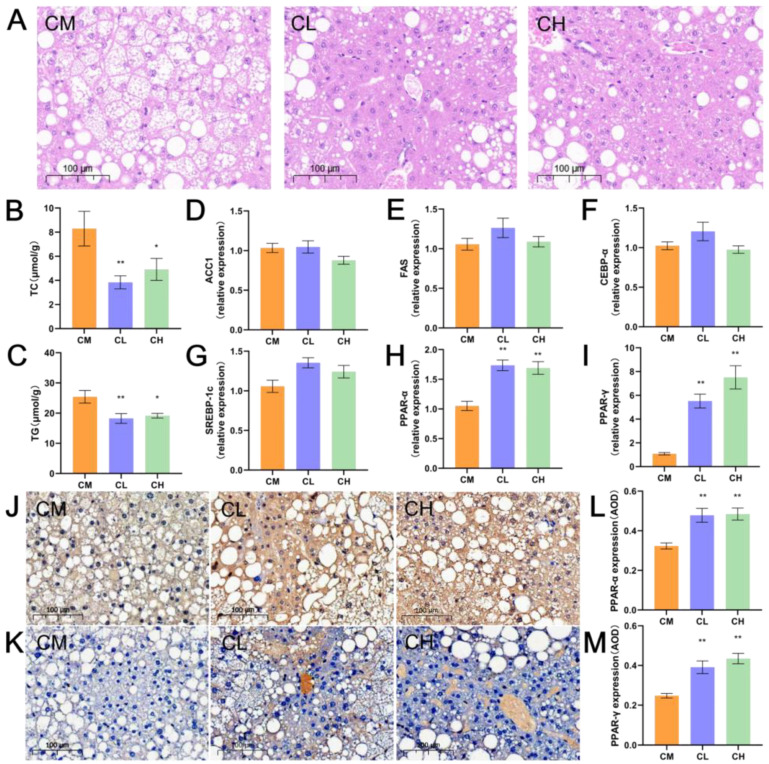
GRP alleviates hepatic lipid accumulation and restores the expression of PPAR-α and PPAR-γ in female *db*/*db* mice. (**A**) Representative H&E staining in the Liver (magnification: 100×, scale: 100 μm). (**B**,**C**) Liver levels of TC, TG. (**D**–**I**) Expression levels of ACC1, FAS, CREBP-α, SREBP-1c, PPAR-α and PPAR-γ mRNA in liver. (**J**,**K**) PPAR-α and PPAR-γ IHC staining of Liver tissue (magnification: 100×, scale: 100 μm). (**L**,**M**) Liver of PPAR-α and PPAR-γ protein AOD value. (* *p* < 0.05, ** *p* < 0.01, Compared with CM group).

Obesity associated with T2DM leads to hepatic fat accumulation. Based on the previously observed modest improvement in serum lipids of female *db*/*db* mice by GRP, we conducted morphological analysis of the liver and measured the content of hepatic TC and TG to further clarify this ameliorative effect and its underlying regulatory pathways. H&E staining revealed marked hepatic steatosis in the CM group, characterized by numerous large cytoplasmic lipid droplets. In contrast, GRP treatment (CL, CH groups) notably reduced steatosis and improved hepatocyte morphology (Figure 3A). Consistent with these histological findings, hepatic TC and TG levels were significantly lower in GRP-treated mice, with reductions of 53.68% and 40.77% in TC, and 28.22% and 24.64% in TG, respectively (Figure 3B,C). These results demonstrate that GRP effectively alleviates hepatic lipid accumulation and improves lipid metabolism in female *db*/*db* mice.

### 2.4. GRP Upregulates Hepatic PPAR-α and PPAR-γ Gene Expression in Female db/db Mice

Given the observed reduction in hepatic lipid content, we further assessed key lipid metabolism genes via RT-qPCR. GRP treatment did not significantly alter the expression of CCAAT/Enhancer-Binding Protein α (CEBP α), Sterol Regulatory Element-Binding Protein 1c (SREBP 1c), Acetyl-Coenzyme A Carboxylase 1 (ACC1), and Fatty Acid Synthase (FAS) (Figure 3D,G), but markedly increased the mRNA levels of PPAR α and PPAR γ (Figure 3H,I). This upregulation was confirmed at the protein level by immunohistochemistry, which showed stronger staining intensity and higher average optical density (AOD) values for both PPAR α and PPAR γ in GRP treated groups compared to the model group (Figure 3J,M). These results indicate that GRP alleviates hepatic lipid deposition primarily through activation of the PPAR signaling axis.

### 2.5. GRP Ameliorates Renal Damage in Female db/db Mice

**Figure 4:**
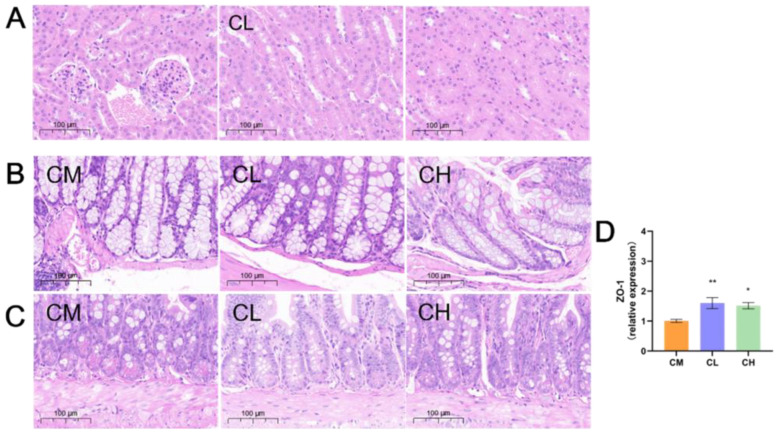
GRP ameliorates renal, colonic, and ileal damage induced by T2DM and restores ZO-1 ex-pression in female *db*/*db* mice. (**A**) Representative H&E staining in the kidney (100×). (**B**,**C**) Representative H&E staining in the colon and ileum (100×). (**D**) Expression levels of ZO-1 mRNA in colon. (* *p* < 0.05, ** *p* < 0.01, Compared with CM group).

T2DM induces organ damage and pathology by exposing tissues and systems to prolonged hyperglycemia, with kidney injury being particularly prominent. Renal H&E staining revealed structural damage in the CM group, including mesangial hyperplasia, widened mesangial areas, focal inflammatory cell infiltration, and tubular vacuolar degeneration. These pathological changes were markedly attenuated in the GRP-treated (CL, CH) groups, which exhibited reduced inflammation and more normalized parenchymal architecture (Figure 4A), indicating that GRP mitigates renal injury in female *db*/*db* mice.

### 2.6. GRP Improves Intestinal Morphology and Upregulates ZO-1 Expression in Female db/db Mice

Given that T2DM induces not only organ damage but also intestinal barrier dysfunction, we performed morphological analysis of the colon and ileum and examined the expression of the tight junction protein Zonula Occludens-1 (ZO-1) in the colon of female *db*/*db* mice to investigate the repairing effect of GRP on the intestinal barrier. H&E staining revealed that GRP treatment ameliorated intestinal damage in female *db*/*db* mice, as evidenced by reduced mucosal inflammation and more regular, intact villi structure in both the colon and ileum compared to the model group (Figure 4B,C). Consistent with this morphological improvement, GRP administration significantly increased the expression of the tight junction protein ZO-1 in the colon (1.6-fold and 1.52-fold in CL and CH groups, respectively; Figure 4D), indicating enhanced intestinal barrier integrity.

### 2.7. GRP Ameliorates Intestinal Dysbiosis in Female db/db Mice

Given that polysaccharides can significantly modulate the gut microbiota, we investigated the regulatory effects of GRP by analyzing the diversity and composition of the bacterial community in the cecal contents of female *db*/*db* mice.

**Figure 5:**
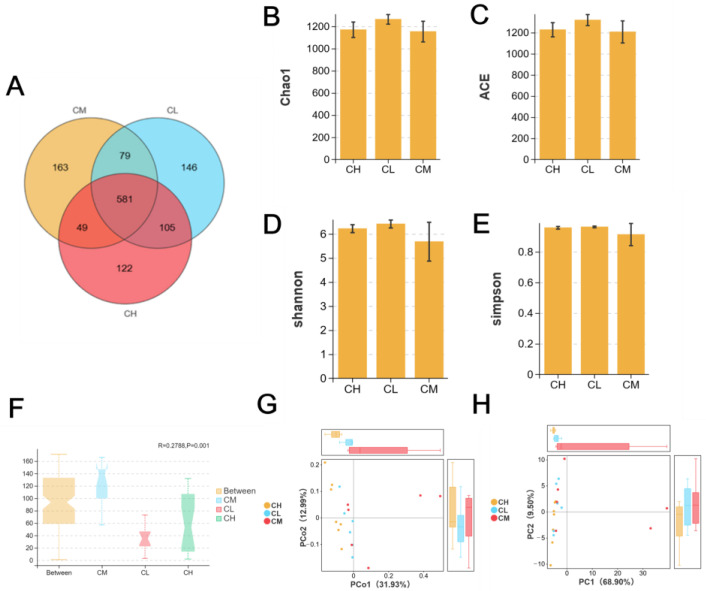
Effects of GRP on intestinal microbial diversity in mice. (**A**) Venn diagram of mouse gut microbiota. (**B**–**E**) α analysis of the indexes of Chao1, ACE, Shannon, Simpson. (**F**–**H**) β analysis of the indexes of Anosim, PCoA, PCA.

Regarding microbial diversity, Analysis of shared Operational Taxonomic Units (OTUs) revealed distinct clustering among groups. While 105 OTUs overlapped between the CL and CH groups, the CM group shared only 79 and 49 OTUs with the CL and CH groups, respectively (Figure 5A), indicating a GRP concentration-dependent shift in microbiota composition. Alpha diversity indices (Chao1, Ace, Shannon, Simpson) showed a reduction in both richness and diversity in GRP-treated groups compared to the CM group (Figure 5B–E), suggesting a selective enrichment of specific taxa rather than a general increase in diversity. Beta diversity analysis (Anosim, PCoA, PCA) confirmed significant structural differences between groups (R = 0.2788, *p* = 0.001; Figure 5F–H), demonstrating that GRP administration substantially altered the overall architecture of the gut microbial community.

**Figure 6:**
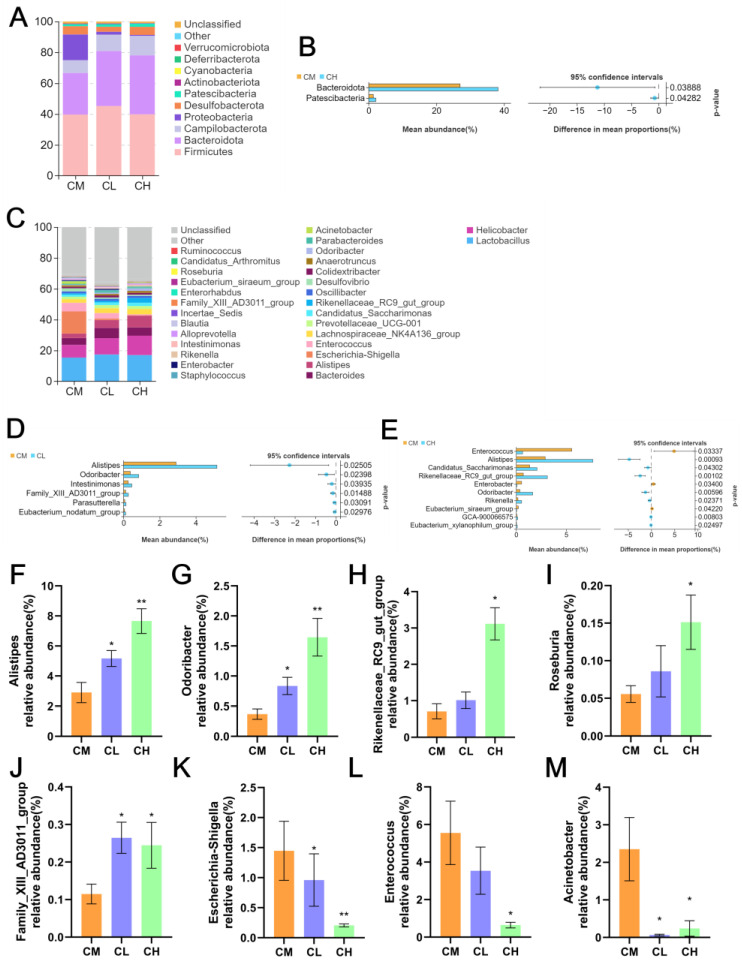
Effects of GRP on species composition and differential species of intestinal flora in female *db*/*db* mice. (**A**) A bar plot of the community at the phylum level. (**B**) Multi-group comparison chart at the phylum level. (**C**) Bar plot of the community at the genus level. (**D**,**E**) Multi-group comparison chart at the genus level. (**F**–**M**) selected bacteria at the genus level. (* *p* < 0.05, ** *p* < 0.01, Compared with CM group).

Regarding microbial composition, At the phylum level (Figure 6A), Firmicutes, Bacteroidetes, Proteobacteria, and Campilobacterota were the dominant phyla (>90% of total bacteria). GRP treatment increased the abundances of *Bacteroidetes* and *Patescibacteria* while decreasing *Proteobacteria*, with these changes being significant in the CH group (Figure 6B). At the genus level (Figure 6C), *Lactobacillus*, *Helicobacter*, and *Bacteroides* were dominant (~30% of total bacteria). GRP-treated groups (CL, CH) showed increased abundances of beneficial genera including *Alistipes*, *Odoribacter*, *Intestinimonas*, *Family_XIII_AD3011_group*, *Candidatus_Saccharimonas*, and *Rikenellaceae_RC9_gut_group* (Figure 6D,E), while the CH group exhibited decreased *Enterococcus*, *Enterobacter*, and *Eubacterium_siraeum_group*. The consistent increase in *Alistipes* and *Odoribacter*, decrease in *Escherichia-Shigella* (Figure 6F–M) suggest these taxa may be key mediators of GRP’s anti-T2DM effects.

**Figure 7:**
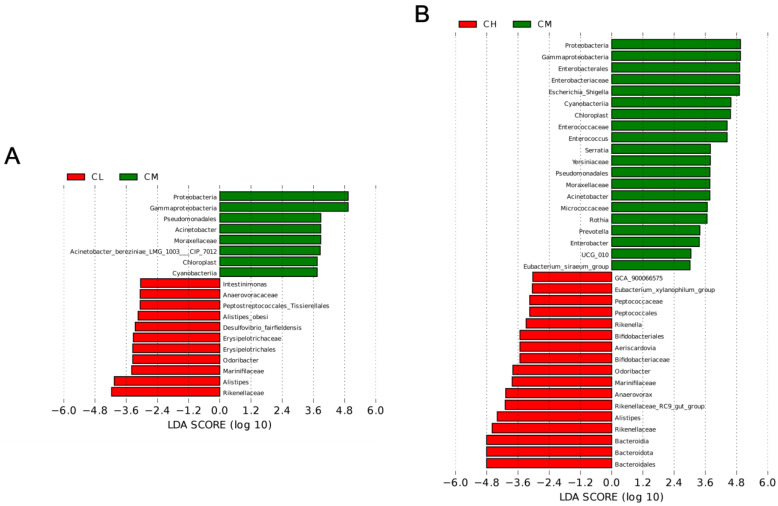
LEfSe analysis of gut microbiota in female *db*/*db* mice. (**A**) Analysis of LEfSe differences in gut microbiota between CL group and CM group. (**B**) Analysis of LEfSe differences in gut microbiota between CH group and CM group.

LEfSe analysis (LDA > 3.0) identified 16 and 37 differentially abundant taxa in the CL and CH groups, respectively (Figure 7A,B). At the phylum level, *Bacteroidota* increased and *Proteobacteria* decreased; at the genus level, *Alistipes* and *Odoribacter* increased, while *Escherichia_Shigella* and *Acinetobacter* decreased. These results further support that GRP alleviates T2DM by selectively modulating specific gut microbiota to improve dysbiosis.

### 2.8. GRP Modulates Kyoto Encyclopedia of Genes and Genomes (KEGG) Functional Pathways

**Figure 8:**
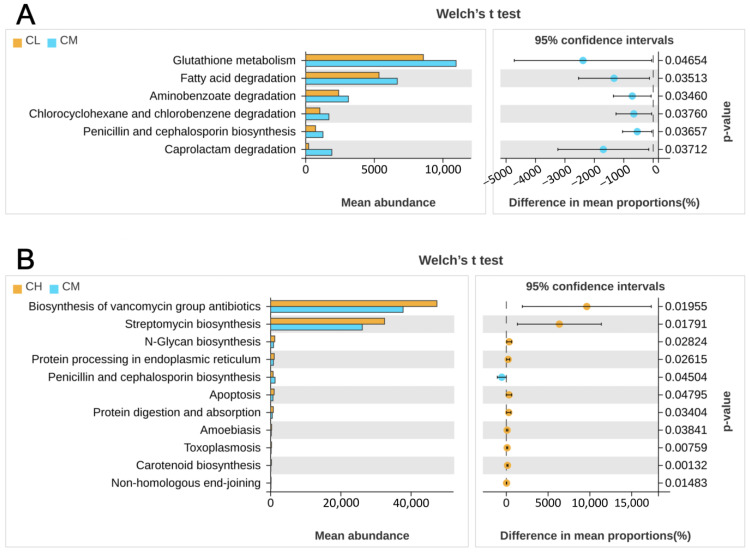
KEGG functional analysis of metabolic pathways between female experimental groups at Level_3 level. (**A**) analysis of inter group differences between CL and CM groups. (**B**) analysis of inter group differences between CH and CM groups.

Based on alterations in microbial composition, KEGG analysis of 16S rRNA sequences was employed to infer potential changes in the community’s collective metabolic functions. Sequences annotated 158 pathways at Level 3. Differential analysis identified 17 pathways significantly affected by GRP, 8 of which were metabolism-related. Compared to the CM group, both CL and CH groups showed significantly reduced functional abundance in the “Penicillin and cephalosporin biosynthesis” pathway (Figure 8A,B). In contrast, the CH group exhibited a significant increase in the “*N*-Glycan biosynthesis” pathway, which is involved in carbohydrate metabolism. These results suggest that GRP may ameliorate T2DM by downregulating penicillin/cephalosporin biosynthesis and upregulating *N*-glycan biosynthesis.

### 2.9. Correlation Analysis Between Biochemical Parameters, Gene Expression, and Gut Microbiota Changes in db/db Mice

**Figure 9:**
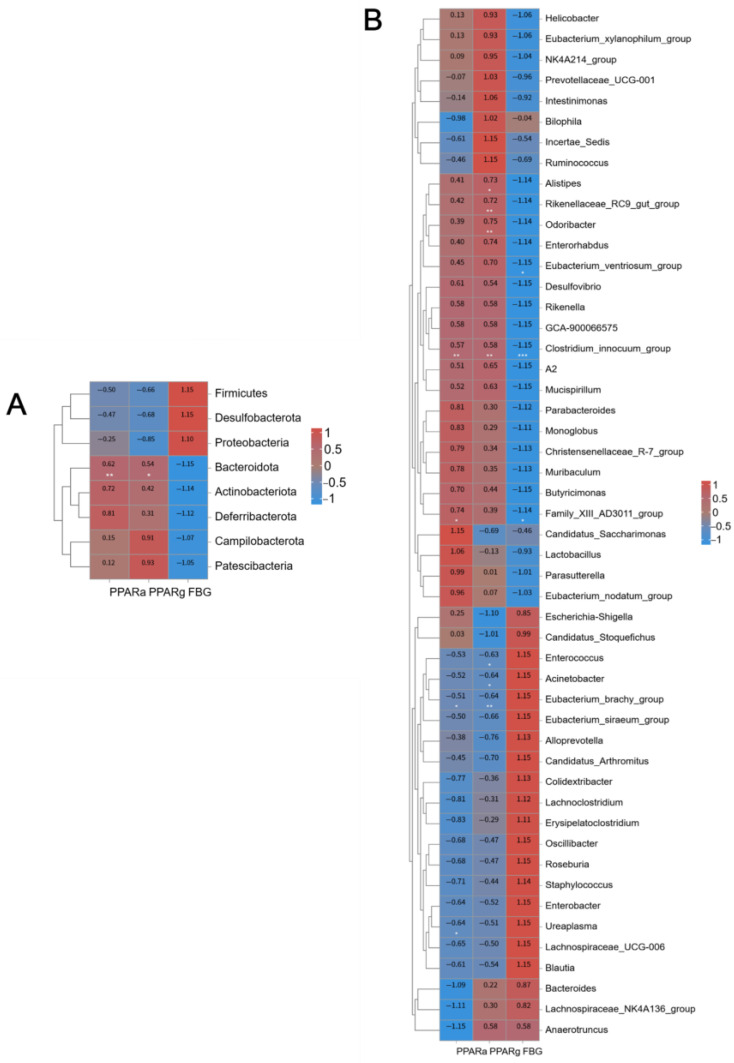
Spearman correlation coefficient heatmap of biochemical indicators, gene expression levels, and gut microbiota in mice. (**A**) correlation analysis at the phylum level. (**B**) correlation analysis at the genus level. (Red represents positive correlation, blue represents negative correlation, darker colors indicate stronger correlation; * *p* < 0.05; ** *p* < 0.01; *** *p* < 0.001).

To explore the functional links between gut microbiota, PPAR α/γ expression, and glucose/lipid metabolism, Spearman correlation analysis was performed. At the phylum level (Figure 9A), *Bacteroidetes* correlated negatively with FBG and positively with PPAR α/γ expression. Conversely, *Proteobacteria*, *Desulfobacterota* and *Firmicutes* showed positive correlations with FBG and negative correlations with PPAR α/γ.

At the genus level (Figure 9B), beneficial genera such as *Alistipes*, *Odoribacter*, *Rikenellaceae_RC9_gut_group*, *Eubacte-rium_ventriosum_group*, *Clostridium_innocuum_group*, *Family_XIII_AD3011_group* and *Roseburia* were negatively correlated with FBG and positively correlated with PPAR α/γ. In contrast, *Escherichia Shigella*, *Eubacterium_brachy_group*, *Enterococcus* and *Acinetobacter* exhibited opposite correlation patterns.

## 3. Discussion

T2DM is a chronic metabolic disorder characterized by hyperglycemia, lipid accumulation, and hepatic/renal injury, largely due to inadequate insulin secretion. Emerging evidence highlights the gut microbiota as a key regulator of T2DM pathogenesis [26]. This study evaluated the effects of GRP on hyperglycemia, organ injury, and gut barrier function in female *db*/*db* mice, and explored its modulation of the gut microbiota.

GRP, composed mainly of polysaccharides (73.48 ± 2.85%), primarily contains glucose, galactose, arabinose, and galacturonic acid, with weight-average molecular weight (M_w_) of 4.711 × 10^6^ Da (Figure 1, Table S3), is similar to the GRP extracted by Liu in terms of monosaccharide composition. Polysaccharides are known for diverse bioactivities and potential in managing metabolic diseases [27]. Studies have shown that polysaccharides exert their effects through multiple signaling and metabolic pathways, playing a role in ameliorating insulin resistance, enhancing glucose uptake, and increasing the production of short-chain fatty acids (SCFAs) [18,28,29,30].

*Db*/*db* mice, bearing a leptin receptor mutation, develop obesity and hyperglycemia from 6 weeks, progressing to full T2DM by 8 weeks [31]. While most studies use males, research on polysaccharides in female *db*/*db* mice remains limited. Hence, we treated 12-week-old female *db*/*db* mice with GRP to assess its effects. GRP administration significantly lowered fasting blood glucose and improved insulin sensitivity in female *db*/*db* mice (Figure 2D,E). T2DM often coexists with dyslipidemia. Studies have shown that dyslipidemia is characterized by elevated serum concentrations of low-density lipoprotein cholesterol (LDL-C), very-low-density lipoprotein cholesterol (VLDL-C), and triglycerides (TG), along with decreased levels of high-density lipoprotein cholesterol (HDL-C) [32]. GRP administration elevated serum HDL-c levels, indicating a modest improvement in the blood lipid profile, although other serum lipids (TC, TG, LDL-c) remained unchanged (Figure 2F–J). GRP significantly reduced hepatic TC and TG content (Figure 3B,C), indicating ameliorated hepatic lipid deposition. The discrepancy with unchanged serum lipid levels might be attributed to: (1) the experimental design involving overnight fasting [33]; and (2) the specific pathway of GRP action. GRP likely activates hepatic lipid catabolic pathways, such as the PPAR signaling axis, enhancing fatty acid oxidation or cholesterol conversion to bile acids, thereby reducing intrahepatic lipid storage without immediately altering systemic circulation [29]. Additionally, we observed that the CL and CH groups did not exhibit a consistent dose-dependent pattern across several parameters, including fasting blood glucose. We propose that this may be attributed to the following reasons: (1) the 300 mg/kg dose may have approached or reached the maximal effect plateau of GRP in improving blood glucose, such that the 600 mg/kg dose did not produce a significantly enhanced effect; (2) GRP may act through multi-target and multi-pathway mechanisms (e.g., modulating gut microbiota, activating PPAR signaling), and its dose-effect relationship may not follow a simple linear pattern. Further studies are warranted to investigate this phenomenon.

GRP administration did not significantly alter the hepatic mRNA expression of key lipogenic genes (*C/EBP-α*, SREBP-1c, *ACC1* and *FAS*) (Figure 3D–G), suggesting this pathway is not its primary mechanism. Instead, GRP increased hepatic PPAR-α and PPAR-γ expression (Figure 3H–M). PPARs are crucial transcription factors maintaining energy balance, glucose/lipid metabolism, and insulin sensitivity. PPAR-α is a key regulator of lipid metabolism, contributing to the reduction in triglycerides and amelioration of hepatic steatosis [34]. In contrast, PPAR-γ enhances systemic insulin sensitivity, promotes adipocyte differentiation, and facilitates glucose uptake, thereby lowering blood glucose levels [35]. More importantly, co-activation of PPAR-α and PPAR-γ produces synergistic effects, offering comprehensive improvement in T2DM by concurrently addressing hyperglycemia and dyslipidemia [36]. In this study, GRP administration in female *db*/*db* mice resulted in lowered blood glucose, improved insulin sensitivity, reduced hepatic steatosis, and upregulated hepatic expression of both PPAR-α and PPAR-γ. These coordinated effects strongly suggest that the therapeutic benefits of GRP against T2DM are mediated, at least in part, through activation of the PPAR signaling axis. Discovering PPAR-α/γ activators from natural extracts is a research focus, as exemplified by *Nigella sativa* extract targeting PPAR-α [37].

As a chronic condition, long-term hyperglycemia in T2DM causes multi-organ damage [5]. GRP treatment alleviated T2DM symptoms and reduced damage in the liver (Figure 3A–C), kidney (Figure 4A), and intestinal (Figure 4B,C) tissues. Upon intestinal barrier damage, ZO-1 orchestrates the balance between epithelial cell apoptosis and proliferation. Its deficiency results in impaired mucosal repair, underscoring its pivotal role in intestinal restitution [38,39]. GRP administration also increased ZO-1 gene expression in the colon (Figure 4D), suggesting enhanced intestinal barrier integrity. GRP treatment improved overall gut health. Microbiota analysis revealed that GRP increased the abundances of beneficial genera like *Alistipes*, *Odoribacter*, *Family_XIII_AD3011_group*, and *Roseburia*, while decreasing harmful genera like *Escherichia-Shigella* and *Acinetobacter*. These genera have established links to glycemic control. *Alistipes* is often dominant in insulin-sensitive individuals, consumes monosaccharides, produces SCFAs, and its restoration can improve insulin resistance [40,41,42,43]. *Odoribacter* ferments carbohydrates to produce butyrate, activating AMPK and enhancing gut barrier function [44,45,46]. *Roseburia* promotes SCFA production, maintains intestinal barrier integrity, and alleviates metabolic disorders [47,48,49,50,51]. *Family_XIII_AD3011_group* abundance is associated with improved metabolic homeostasis and reduced inflammation [52,53]. Conversely, *Escherichia-Shigella* abundance correlates with colonic inflammation, promotes endotoxin production, and activates inflammatory pathways in T2DM patients [54,55,56,57]. *Acinetobacter* is linked to increased inflammation and complications in T2DM, and its reduction may improve metabolic status [58,59]. GRP treatment reshaped the gut microbiota structure in a beneficial manner, characterized by a concerted increase in SCFA-producing genera (e.g., *Alistipes*, *Odoribacter*, *Roseburia*) and a reduction in inflammation-associated genera (e.g., *Escherichia-Shigella*, *Acinetobacter*). This shift is functionally significant: the enriched beneficial bacteria contribute to improved intestinal barrier integrity, enhanced insulin sensitivity, and anti-inflammatory effects, while the suppressed harmful bacteria are linked to decreased endotoxin production and colonic inflammation. Collectively, these microbiota alterations underpin the observed improvement in overall gut health and glycemic control, highlighting a key mechanism through which GRP may ameliorate T2DM.

This study presents the first report that, in female *db*/*db* mice, *Glehniae Radix* polysaccharides exert hypoglycemic effects, enhance insulin sensitivity, and improve intestinal health by modulating the abundance of specific gut microbiota—promoting beneficial genera such as *Alistipes* and *Odoribacter* while suppressing harmful ones—and by activating the PPAR signaling axis.

This study has several limitations that should be acknowledged. (1) GRP is a crude polysaccharide extract derived from *Glehniae Radix*; therefore, its specific active constituents have not yet been identified. Future studies will focus on further isolation and purification to validate the efficacy of individual components. (2) A clear dose–response relationship was not observed for certain parameters (e.g., ZO-1 expression). It is speculated that the 300 mg/kg dose of GRP may have already reached the maximal effect plateau for diabetes amelioration. Subsequent research should employ narrower dose intervals to further investigate the potential dose–response relationship. (3) This study did not employ reverse validation experiments such as PPAR antagonists or fecal microbiota transplantation (FMT); therefore, the causal mechanism requires further confirmation. (4) This study did not experimentally validate the specific roles of GRP-modulated bacterial genera (such as *Alistipes* or *Odoribacter*), leaving the precise microbial drivers within the “gut microbiota-PPAR signaling axis” unresolved. (5) The sample size was limited, contributing to considerable individual variability in certain parameters. (6) A male control group was not included; thus, the influence of sex differences remains unclear. The above considerations provide direction for future in-depth research, and subsequent studies should aim to address these gaps.

## 4. Materials and Methods

### 4.1. Chemicals and Reagents

The chemicals used, including anhydrous ethanol, chloroform, and isopropanol, were purchased from Guangdong Xilong Scientific Co., Ltd. (Shantou, China). TRIzol reagent was from Thermo Fisher Scientific Inc. (Waltham, MA, USA). Diethyl pyrocarbonate (DEPC)-treated water was obtained from SenBeiJia Biological Technology Co., Ltd. (Nanjing, China). Prime-Script™ RT Master Mix was from Takara Biomedical Technology Co., Ltd. (Beijing, China). 2× SYBR GREEN qPCR Mix was from Xiamen Kangji Biotechnology Co., Ltd. (Xiamen, China).

### 4.2. Plant Materials and Preparation of the GRP

Dried roots of *Glehnia littoralis* (*Glehniae Radix*): Shandong Province origin, Lot No. 230601. GRP was extracted using the hot water extraction method. Briefly, 200 g of powdered *Glehniae Radix* was mixed with 6000 mL of purified water (solid-to-liquid ratio = 1:30 g/mL). The mixture was heated at 60 °C for 100 min. After extraction, the solution was centrifuged at 6000 r/min for 10 min, and the supernatant was collected. The residue was re-extracted under identical conditions with another 6000 mL of purified water. The combined supernatants were concentrated to 1200 mL. Four volumes of 95% ethanol were added gradually with stirring, and the mixture was stored at 4 °C overnight. The resulting precipitate was collected by centrifugation at 6000 r/min for 10 min, followed by freeze-drying to obtain the GRP.

### 4.3. Chemical Composition and Structural Characterization of GRP

The total polysaccharide content in GRP was determined using the phenol-sulfuric acid method with D-glucose as the standard. The total uronic acid content was measured by the carbazole-sulfuric acid method using galacturonic acid as the standard. The total protein content was quantified using the bicinchoninic acid (BCA) assay with bovine serum albumin as the standard. The molecular weight distribution of GRP was analyzed by High-Performance Size-Exclusion Chromatography coupled with Multi-Angle Laser Light Scattering (SEC-MALS). The analysis was performed using a TSKgel GMPWXL column (7.8 × 300 mm) with 0.2 mol/L sodium nitrate solution as the mobile phase at a flow rate of 0.5 mL/min and a column temperature of 35 °C. Detection was carried out using an 18-angle laser photometer and a refractive index detector. Fourier Transform Infrared (FT-IR) spectroscopy was performed using the potassium bromide (KBr) pellet method. Briefly, 2 mg of GRP was mixed with 100 mg of KBr and pressed into a pellet. The FT-IR spectrum was recorded in the range of 400–4000 cm^−1^. The monosaccharide composition of GRP was determined by acid hydrolysis followed by pre-column derivatization with 1-phenyl-3-methyl-5-pyrazolone (PMP) and subsequent High-Performance Liquid Chromatography (HPLC) analysis. The analysis was conducted on a Thermo Fisher Scientific UltiMate 3000 HPLC system (Waltham, MA, USA) equipped with a Thermo C18 column (4.6 × 250 mm) with a mobile phase consisting of acetonitrile and 0.1 mol/L phosphate buffer (pH 7.0) (17:83, *v*/*v*) at a flow rate of 0.8 mL/min. The column temperature was maintained at 30 °C, and detection was performed using a UV detector at 254 nm.

### 4.4. Animal Experimentation

Twenty-four 12-week-old female B6.BKS(D)-Leprdb/J (*db*/*db*) mice with a leptin receptor mutation, bred in the Specific Pathogen-Free (SPF) animal facility of the Third Institute of Oceanography, Ministry of Natural Resources, were used. The *db*/*db* mice were randomly divided into three groups (*n* = 8 per group): the model group (untreated) and two GRP-treated groups. The treated groups received daily oral gavage of GRP at doses of 300 or 600 mg/kg body weight, respectively, for 5 weeks, while the model group received an equivalent volume of ultrapure water. The body weight and average food intake of mice in each group were recorded daily. At the end of the experimental period, the mice were euthanized. Serum, liver, kidney, colon, ileum tissues, and cecal contents were collected for Hematoxylin and Eosin (H&E) staining analysis and stored at −80 °C for subsequent analysis. All animal experimental protocols were reviewed and approved by the Animal Ethics Committee of the Third Institute of Oceanography, Ministry of Natural Resources (Approval No. TIO-IACUC-01-2025-03-27).

### 4.5. Body Weight, Food Intake, Fasting Blood Glucose, and Insulin Tolerance Test

Body weight and food intake were recorded weekly throughout the 5-week experimental period. Fasting blood glucose levels were measured in the third week. An insulin tolerance test (ITT) was performed in the fifth week. After fasting, mice were intraperitoneally injected with insulin at a dose of 0.75 U/kg body weight. Blood glucose levels were measured at 0, 30, 60, and 120 min post-injection.

### 4.6. Biochemical Parameter Analysis in Serum and Liver

Serum levels of triglycerides (TG), total cholesterol (TC), high-density lipoprotein cholesterol (HDL-c), and low-density lipoprotein cholesterol (LDL-c) were determined using assay kits purchased from Shenzhen Mindray Bio-Medical Electronics Co., Ltd. (Shenzhen, China). The analyses were performed on an automatic biochemical analyzer according to the manufacturer’s (Mindray, Shenzhen, China) protocols.

Hepatic TC and TG levels in mice were measured using the corresponding assay kits provided by the Nanjing Jiancheng Bioengineering Institute (Nanjing, China), following the manufacturer’s guidelines.

### 4.7. Histopathological Analysis

Fresh liver, kidney, colon, and ileum tissues were collected from mice and fixed in 10% neutral buffered formalin (tissue fixative solution from Guangzhou VIGGS Biotechnology Co., Ltd., Guangzhou, China) to preserve tissue architecture by denaturing and coagulating proteins, thereby preventing bacterial degradation and autolysis. Subsequently, 4-μm paraffin sections were prepared. After deparaffinization and rehydration, the sections were stained with Hematoxylin and Eosin (H&E), followed by dehydration and mounting. Histopathological changes were examined under a light microscope (ZEISS, Oberkochen, Germany) at 100× magnification.

### 4.8. RNA Isolation and Quantitative Real-Time Polymerase Chain Reaction (RT-qPCR)

Total RNA was extracted from approximately 50 mg of liver or colon tissue using TRIzol reagent. The concentration and purity of the isolated RNA were determined using a microvolume spectrophotometer. Subsequently, 2 µg of total RNA was reverse-transcribed into cDNA using the PrimeScript™ RT Master Mix on a thermal cycler (Takara Bio, San Jose, CA, USA), under the following conditions: 37 °C for 15 min followed by enzyme inactivation at 95 °C for 5 s. Quantitative PCR was performed using the 2× SYBR GREEN qPCR Mix on a real-time PCR detection system. The primer sequences used are listed in Table S1. The PCR amplification protocol consisted of an initial denaturation at 95 °C for 5 min, followed by 40 cycles of denaturation at 95 °C for 30 s, annealing at 56 °C for 30 s, and extension at 72 °C for 45 s. A melt curve analysis was performed subsequently (95 °C for 10 s, 65 °C for 60 s, and 97 °C for 1 s). Relative gene expression levels were calculated using the 2^−ΔΔCT^ method, normalized to GAPDH as housekeeping gene and expressed as fold change relative to the model group.

### 4.9. Immunohistochemical (IHC) Staining of Mouse Liver Tissue

IHC staining was performed to evaluate the protein expression levels of proliferator-activated receptor-α (PPAR-α) and proliferator-activated receptor-γ (PPAR-γ) in liver tissues. Formalin-fixed liver specimens were embedded in paraffin and sectioned. The sections were then deparaffinized, rehydrated, and subjected to antigen retrieval. Endogenous peroxidase activity was blocked by incubation with 3% hydrogen peroxide. Following antigen retrieval, the sections were incubated with mouse primary antibodies specific for PPAR-α and PPAR-γ. Cell nuclei were counterstained with hematoxylin, after which the sections were dehydrated, cleared, and mounted. Stained sections were examined under a microscope, and images were quantitatively analyzed using ImageJ software 2.14.0. Protein expression levels were determined based on the average optical density (AOD).

### 4.10. Analysis of Mouse Gut Microbiota

Mouse intestines were aseptically dissected, and the cecal contents were collected under sterile conditions. Gut microbiota sequencing was performed by Guangzhou Gidio Biotechnology Co., Ltd. (Guangzhou, China). The specific procedures were as follows: Genomic DNA was extracted from the samples. The conserved regions of bacterial rDNA were amplified using barcoded specific primers. The resulting PCR amplicons were purified by gel extraction and quantified using a QuantiFluor™ fluorometer (Promega, Madison, WI, USA). The purified amplicons were pooled in equimolar amounts, and sequencing adapters were ligated to construct the sequencing library. Paired-end (2 × 250 bp) sequencing was performed on an Illumina platform, generating raw sequencing data. Bioinformatic analysis of the mouse gut microbiota was conducted using Omicsmart, a dynamic real-time interactive online platform for data analysis.

### 4.11. Data Processing

All raw data were collated using Microsoft Excel 2013. Graphs were generated using GraphPad Prism 8.3.0 (Graphpad Software, Inc., Boston, MA, USA). Data are presented as the mean ± standard error of the mean (SEM). Given the relatively small sample sizes, the Shapiro–Wilk test was specifically employed to assess normality. The results showed that the *p*-values for all tested groups were >0.05, indicating that the data conform to a normal distribution. Statistical comparisons between groups were performed using one-way analysis of variance (ANOVA). A *p*-value of less than 0.05 was considered statistically significant.

## 5. Conclusions

This study demonstrates that GRP is a crude polysaccharide predominantly composed of glucose, with a total polysaccharide content of 73.48% and an acidic sugar content of 46.09%. Animal experiments revealed that GRP administration significantly reduced fasting blood glucose levels, enhanced insulin sensitivity, and increased serum HDL-C levels in female *db*/*db* mice. Histopathological analysis indicated that GRP ameliorated tissue lesions in the liver and kidney and markedly decreased hepatic TC and TG content. IHC and PCR results demonstrated that GRP significantly increased both mRNA and protein expression of PPAR-α and PPAR-γ in the liver. Concurrently, GRP repaired the intestinal barrier in the colon and ileum and increased the expression of ZO-1 in the colon. Analysis of cecal contents via 16S rRNA high-throughput sequencing showed that GRP altered the richness, diversity, and compositional structure of the gut microbiota in female *db*/*db* mice. Specifically, GRP promoted the abundance of beneficial genera, including *Alistipes*, *Odoribacter*, *Family_XIII_AD3011_group*, and *Roseburia*, while suppressing the abundance of potentially harmful genera, such as *Escherichia-Shigella* and *Acinetobacter*. Furthermore, GRP modulated host metabolism by enhancing the *N*-Glycan biosynthesis pathway and inhibiting the Penicillin and cephalosporin biosynthesis pathway, collectively contributing to its hypoglycemic effects. A limitation of this study is the lack of experiments utilizing PPAR antagonists or fecal microbiota transplantation to further validate the mechanism by which GRP ameliorates T2DM in female *db*/*db* mice through the modulation of the gut microbiota and the PPAR signaling axis. This lays the groundwork for future investigations.

## Data Availability

The original contributions presented in this study are included in the article/Supplementary Material. Further inquiries can be directed to the corresponding authors.

## Supplementary Materials

The following supporting information can be downloaded at: https://www.mdpi.com/article/10.3390/molecules31061046/s1. Figure S1. Standard curve. (A) Glucose standard curve, (B) galacturonic acid standard curve, (C) Protein standard curve. Figure S2. Molecular weight detection spectrum of GRP. Table S1. The sequences of primers used in RT-qPCR. Table S2. Molecular weight analysis of GRP. Table S3. Chemical composition of GRP. Table S4. Monosaccharide composition of GRP.

## Abbreviations

The following abbreviations are used in this manuscript:

T2DM	particularly type 2 diabetes
HSHF	high-sugar/high-fat
IR	insulin resistance
SCFAs	short-chain fatty acids
GRPs	*Glehniae Radix* polysaccharides
BCA	bicinchoninic acid
SEC-MALS	Multi-Angle Laser Light Scattering
PMP	1-Phenyl-3-methyl-5-pyrazolone
Man	D-Mannose
GlcN	D-Glucosamine
Rha	L-Rhamnose
GlcA	D-Glucuronic acid
GalA	D-Galacturonic acid
GalN	D-Galactosamine
Glc	D-Glucose
Gal	D-Galactose
Xyl	D-Xylose
Ara	L-Arabinose
Fuc	L-Fucose
FT-IR	Fourier Transform Infrared Spectroscopy
HPLC	High-Performance Liquid Chromatography
*db*/*db*	B6.BKS(D)-Leprdb/J
SPF	Specific Pathogen-Free
FBG	fasting blood glucose
ITT	An insulin tolerance test
AUC	area under the curve
TG	triglyceride
TC	total cholesterol
HDL-c	high-density lipoprotein cholesterol
LDL-c	low-density lipoprotein cholesterol
PCR	Polymerase Chain Reaction
RT-qPCR	Quantitative Real-time polymerase chain reaction
CEBP-α	CCAAT/Enhancer-Binding Protein α
SREBP-1c	Sterol Regulatory Element-Binding Protein 1c
ACC1	Acetyl-Coenzyme A Carboxylase 1
FAS	Fatty Acid Synthase
PPAR-α	proliferator-activated receptor-α
PPAR-γ	proliferator-activated receptor-γ
AOD	average optical density
ZO-1	Zonula Occludens-1
GAPDH	Glyceraldehyde-3-Phosphate Dehydrogenase
OTUs	Operational Taxonomic Units
