# Supplementary material for: Hypoglycemic Effects of Glehniae Radix Polysaccharides in Female db/db Mice via Modulation of the Gut Microbiota-PPAR Signaling Axis
Source: Molecules. 2026 Mar 20;31(6):1046. doi: 10.3390/molecules31061046 (PMC13029656; doi:10.3390/molecules31061046)
Supplement: Supplementary file 1 [file molecules-31-01046-s001.zip › molecules-4181200-supplementary.pdf]

Supplementary Materials

Hypoglycemic Effects of *Glehniae Radix* Polysaccharides in Female *db/db* Mice via Modulation of the Gut Microbiota-PPAR Signaling Axis

Haochen Xu [1,†], Hanqing Lin [1,†], Hetong Lin [1], Peng Wu [2], Fang Zhang [2,*] and Longhe Yang [2,*]

[1] College of Food Science, Fujian Agriculture and Forestry University, Fuzhou 350002, China; xuhaochen1008@163.com (H.X.); papapu.ppa.pu@gmail.com (H.L.); hetonglin@163.com (H.L.)
[2] Technical Innovation Center for Utilization of Marine Biological Resources, Third Institute of Oceanography, Ministry of Natural Resources, Xiamen 361000, China; pwu@tio.org.cn
* Correspondence: fzhang@tio.org.cn (F.Z.); longheyang@tio.org.cn (L.Y.)
† These authors contributed equally to this work.

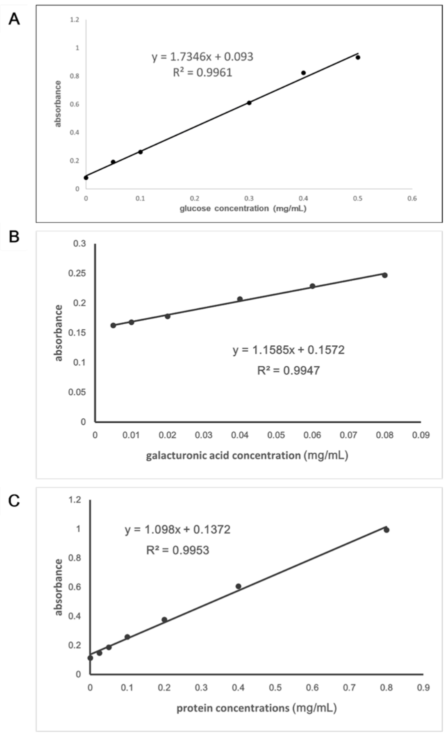

Figure S1. Standard curve. (**A**) Glucose standard curve, (**B**) galacturonic acid standard curve, (**C**) Protein standard curve.

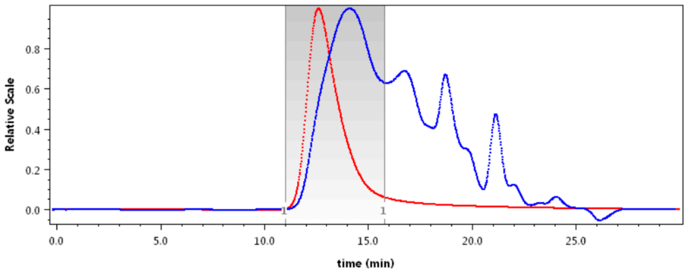

Figure S2. Molecular weight detection spectrum of GRP.

Table S1. The sequences of primers used in RT-qPCR.

Gene	Sequences (5′-3′)		Base Pair (BP)
CEBP-α	Forward	ATAGACATCAGCGCCTACAT	20
	Reverse	TCCCGGGTAGTCAAAGTCAC	20
SREBP-1c	Forward	GGCTGTTGTCTACCATAAGC	20
	Reverse	AGGAAGAAACGTGTCAAGAA	20
ACC1	Forward	CAAGTGCTCAAGTTTGGCGC	20
	Reverse	CAAGAACCACCCCGAAGCTC	20
FAS	Forward	CTGGCATTCGTGATGGAGTC	20
	Reverse	TGTTTCCCCTGAGCCATGTA	20
PPAR-γ	Forward	CTCCAAGAATACCAAAGTGCGA	20
	Reverse	GCCTGATGCTTTATCCCCACA	20
PPAR-α	Forward	TTTCGGCGAACTATTCGGCTG	20
	Reverse	GGCATTTGTTCCGGTTCTTCTT	20
ZO-1	Forward	GAGCGGGCTACCTTACTGAAC	23
	Reverse	GTCATCTCTTTCCGAGGCATTAG	19
GAPDH	Forward	TGACCTCAACTACATGGTCTACA	23
	Reverse	CTTCCCATTCTCGGCCTTG	19

Table S2. Molecular weight analysis of GRP.

Polysaccharides (%)	Uronic Acid (%)	Protein (%)
73.48 ± 2.85	46.09 ± 1.56	0.85 ± 0.05

Table S3. Chemical composition of GRP.

Mn (Da)	Mp (Da)	Mw (Da)	Mw/Mn
1.938×10^6	2.064×10^6	4.711×10^6	2.44

Table S4. Monosaccharide composition of GRP.

Glucose (mol%)	Galactose (mol%)	Arabinose (mol%)	Glucuronic Acid (mol%)	Rhamnose (mol%)	Mannose (mol%)
92.87	2.36	2.28	1.77	0.41	0.30
